# The Influence of Arginine on the Response of Enamel Matrix Derivative (EMD) Proteins to Thermal Stress: Towards Improving the Stability of EMD-Based Products

**DOI:** 10.1371/journal.pone.0144641

**Published:** 2015-12-15

**Authors:** Alessandra Apicella, Peggy Heunemann, Sreenath Bolisetty, Matteo Marascio, Anja Gemperli Graf, Laszlo Garamszegi, Raffaele Mezzenga, Peter Fischer, Christopher J. Plummer, Jan-Anders Månson

**Affiliations:** 1 Laboratoire des Technologie des Composites et Polymères (LTC), Ecole Polytechnique Fédérale de Lausanne (EPFL), 1015 Lausanne, Switzerland; 2 Food Process Engineering, Institute of Food, Nutrition and Health, ETH Zurich, 8092 Zurich, Switzerland; 3 Food and Soft Materials Science, Institute of Food, Nutrition and Health, ETH Zurich, 8092 Zurich, Switzerland; 4 Institut Straumann AG, 4052 Basel, Switzerland; Institute for Bioengineering of Catalonia, SPAIN

## Abstract

In a current procedure for periodontal tissue regeneration, enamel matrix derivative (EMD), which is the active component, is mixed with a propylene glycol alginate (PGA) gel carrier and applied directly to the periodontal defect. Exposure of EMD to physiological conditions then causes it to precipitate. However, environmental changes during manufacture and storage may result in modifications to the conformation of the EMD proteins, and eventually premature phase separation of the gel and a loss in therapeutic effectiveness. The present work relates to efforts to improve the stability of EMD-based formulations such as Emdogain^™^ through the incorporation of arginine, a well-known protein stabilizer, but one that to our knowledge has not so far been considered for this purpose. Representative EMD-buffer solutions with and without arginine were analyzed by 3D-dynamic light scattering, UV-Vis spectroscopy, transmission electron microscopy and Fourier transform infrared spectroscopy at different acidic pH and temperatures, *T*, in order to simulate the effect of pH variations and thermal stress during manufacture and storage. The results provided evidence that arginine may indeed stabilize EMD against irreversible aggregation with respect to variations in pH and *T* under these conditions. Moreover, stopped-flow transmittance measurements indicated arginine addition not to suppress precipitation of EMD from either the buffers or the PGA gel carrier when the pH was raised to 7, a fundamental requirement for dental applications.

## Introduction

Enamel Matrix Derivative (EMD) is an acidic porcine fetal tooth extract made up of over a hundred different extra-cellular proteins (90% amelogenin, 5% ameloblastin, 5% others) [1], and is the active component of a therapeutic gel used in the regenerative treatment of periodontal disease [2–5]. In this formulation, propylene glycol alginate (PGA) acts as a carrier for the deposition of EMD onto the affected tissue [6]. On reaching physiological pH and temperature, *T*, the EMD precipitates to form a compact layer that promotes cell proliferation, periodontal ligament extension and consequent periodontal tissue regeneration [7, 8]. However, as is generally the case with therapeutic proteins, it is important for EMD to remain stable against denaturation and irreversible aggregation during the various stages of manufacture, storage and delivery, if its regenerative capacity is to be maintained.

The principal component of EMD, amelogenin, typically present in monomeric form in aqueous media at low pH, is widely reported to self-assemble into nanosized spherical aggregates with diameters of the order of 10 to 100 nm [9], referred to in what follows as “nanospheres”. These agglomerate further to form three-dimensional networks as the pH is raised towards the isoelectric point of 6.8 [10–19]. Self-assembly is thought to involve hydrophobic interactions, while the size of the resulting aggregates may be limited by the presence of the hydrophilic C-terminal domains in full-length amelogenin [10–20]. Such processes are sensitive not only to pH but also to variations in *T* [21, 22], and the associated changes in conformation and solubility are also expected to have a direct bearing on EMD precipitation during the clinical application of EMD-PGA gel. However, even for amelogenin the precise links between secondary structure and aggregation remain unclear [18].

A straightforward approach to improving the stability of proteins with respect to environmental stress is to combine them with suitable excipients, such as certain amino acids, salts and buffers [23–26]. One of the most widely used additives for protein stabilization is arginine, which is a common naturally occurring amino acid that has been proven to be effective for a comprehensive range of proteins [27–31]. Moreover, arginine is currently used in treatments for dentin hypersensitivity, and its compatibility with dental tissue is well established [32].

In the present work, we have investigated the response of EMD-buffer solutions at strongly and weakly acidic pH to heat treatments representative of those used in the manufacture of EMD-based products and, for the first time to our knowledge, the use of arginine to stabilize its behavior under these conditions. To further assess its suitability for use in formulations for regenerative dental therapy, the influence of arginine on the precipitation of EMD from EMD-buffer and EMD-PGA-buffer solutions at physiological pH has also been studied using rapid kinetics stopped-flow (SF) measurements.

## Experimental Methods

### Materials

EMD (Biora/Straumann, composition as described in detail in reference [33]) was dissolved in 0.1 M Britton-Robinson (BR) buffer (H_3_BO_3_, H_3_PO_4_, CH_3_COOH) at room temperature to give a final concentration of 29 mg/mL, and the pH adjusted to 2 and 5 by varying the concentrations of the individual BR components. Where required, 500 mM of arginine was dissolved in the buffer solutions prior to EMD addition. Homogeneous EMD-PGA-buffer solutions were prepared by adding PGA in the ratio 2 parts PGA to 1 part EMD by weight, and stirring overnight at room temperature. The specimens were stored at 4°C overnight before further testing.

### Dynamic light scattering (DLS)

Advanced modulated 3D cross correlation DLS was used to investigate the EMD aggregate dimensions. The measurements were performed using apparatus from LS Instruments AG, Switzerland, equipped with a He-Ne laser emitting a polarized light beam of wavelength of 632.8 nm. Specimens were placed in 10 mm diameter glass tubes, and measurements performed at *T* from 20 to 70°C in steps of 10°C. A further measurement was then performed after cooling back to 20°C. The sample cell was maintained within +/- 0.1°C of the required *T* using a thermostat. The scattered intensity fluctuations were collected at a fixed angle of 90° for 10 minutes, so that the effective ramp rate during heating was about 0.8°C/min. The results were averaged over three independent runs and the time correlation function of the scattered intensity was analyzed using the CONTIN method [34].

### Transmission Electron Microscopy (TEM)

Specimens were prepared for TEM (Tecnai Spirit BioTWIN in bright field mode with an accelerating voltage of 80 kV) by negative staining using a sequential two-droplet method [35].

### UV-Vis absorption spectroscopy

Aqueous EMD was placed in a quartz cuvette with a 1 mm path length, and the UV-Vis absorbance was evaluated in the wavelength range 250 to 350 nm using a V-670 Varian spectrometer [36]. Temperature *T* was increased from 20 to 80°C in steps of 10°C. Spectra were acquired at each step with a dwell time of 5 minutes to give an overall ramp rate of about 5°C/min. Each absorption spectrum was baseline corrected by subtraction of a 4^th^ order polynomial fit to the background scattering signal.

### Fourier transform (FTIR) infrared spectroscopy

200 μL aliquots from the EMD-buffer solutions were incubated at 20°C for 1 hr. *T* was increased to 80°C at 2°C/min and the aliquots incubated at this *T* for a further 1 hr. They were then cooled to 20°C at 2°C/min and incubated for a further 12 hours to improve their stability. FTIR absorbance measurements were carried out before and after heat treatment by depositing a 10 μL drop of each specimen onto a polished CaF_2_ IR window and letting it dry. Secondary derivative analysis and normalization were used to resolve the fine structure of the spectra in the amide I band (1600–1700 cm^-1^) which is dominated by the C = O stretch vibrations of the peptide linkages.

### Stopped-flow measurements

The release kinetics from the EMD- and EMD-PGA-buffer solutions, and their arginine-modified counterparts were investigated by rapid kinetics stopped-flow measurements, with a BioLogic SFM400/S device connected to a MOS-250 spectrometer unit, emitting light at a wavelength of 290 nm [37]. The pH was increased to 7 during the stopped-flow measurements by addition of 250 mM NaOH to the specimens initially at pH 2 and 50 mM NaOH to the specimens initially at pH 5. A total flow rate of 6 mL/s and total flow cell volume of approximately 351 μL were used throughout. Under these conditions, use of a FC-15 flow cell for the transmittance detection implied a dead time of 10 ms. The buffered specimens and NaOH solutions were stored in high performance syringes and equivalent volumes injected (1:1) via a high-density mixer into the observation flow cell (1.5 mm light path length). The transmittance, *Tr*, was measured over a time, *t*, of approximately 10 s, the regime associated with precipitation kinetics as opposed to sedimentation, which is characterized by longer *t*. Reproducibility was verified by performing the measurements in quadruplicate and the standard deviation in *Tr* was found to be of the order of 2%.

## Results

### Aggregation and conformational behavior of the EMD

**Fig 1:**
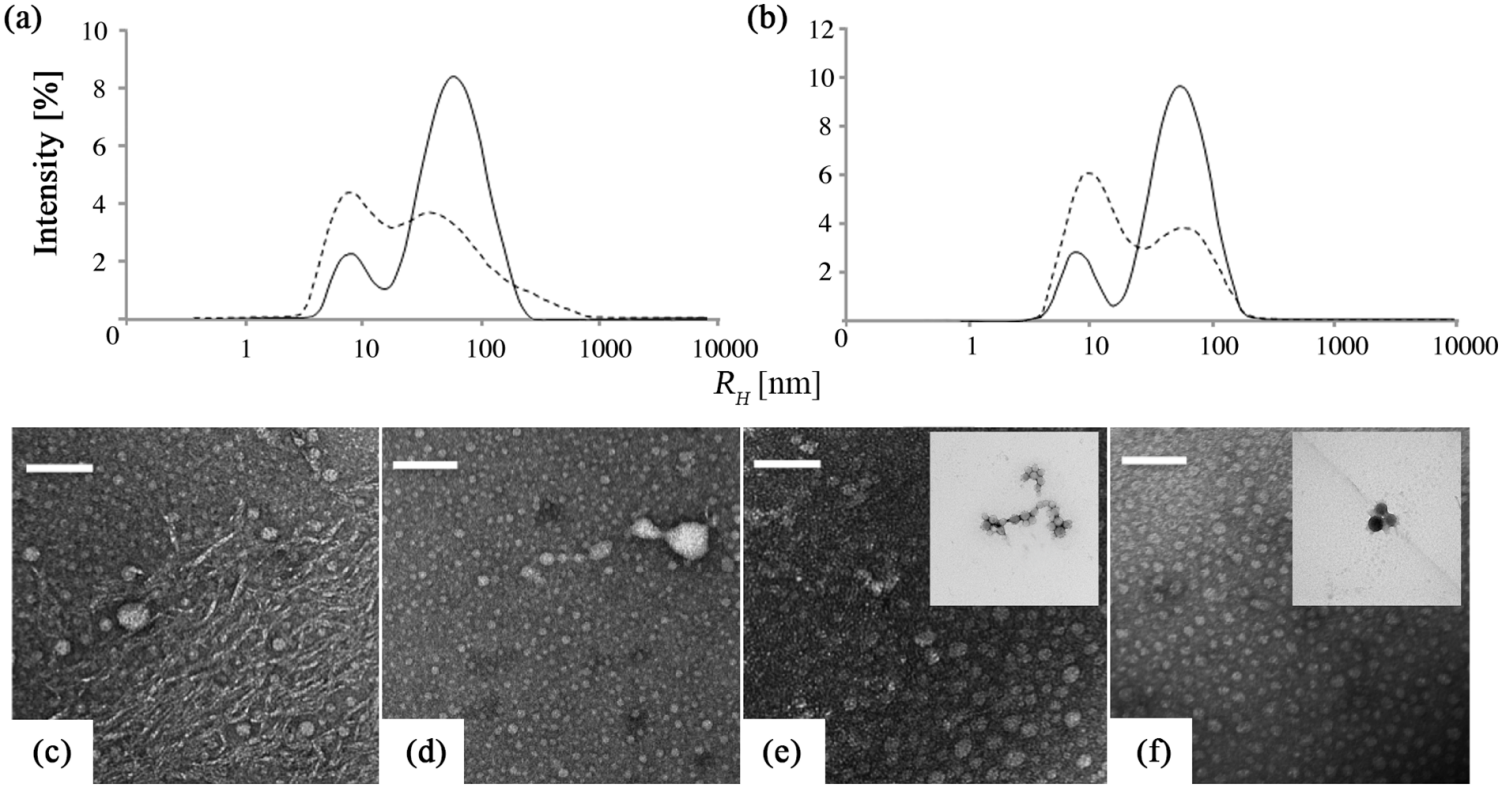
3D-DLS size distributions for EMD at pH 2 and 20°C, (a) without and (b) with arginine, before (solid curve) and after (hatched curve) heat treatment at 70°C. TEM micrographs of EMD at pH 2 and 20°C without arginine, (c) before and (d) after heat treatment. TEM micrographs of EMD at pH 2 and 20°C with arginine, (e) before and (f) after heat treatment. The scale bar corresponds to 50 nm in all the images and the insets in (e) and (f) show examples of relatively large agglomerates also present in these specimens.

**Fig 2:**
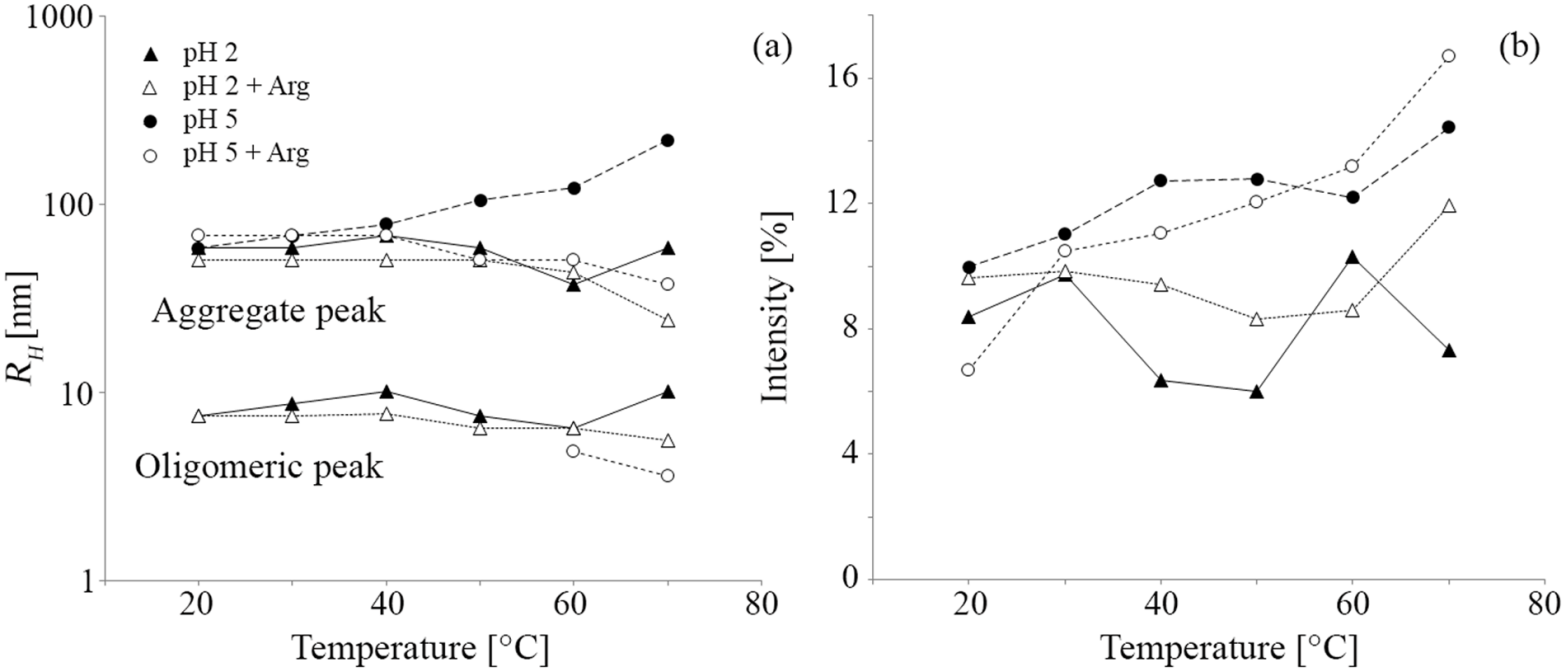
*T* dependence of the DLS data: (a) positions of the oligomeric and aggregate peaks during heating; (b) intensity of the aggregate peak.

The EMD particle size distribution in BR buffer at pH 2 obtained by DLS showed peaks at approximately 8 and 60 nm prior to heat treatment, taken to correspond to oligomers and a distinct population of larger soluble aggregates, respectively (Fig 1(a)). After *T* was raised to 70°C and then reduced to 20°C, the size distribution broadened to include hydrodynamic radii, *R*_*H*_, of up to several hundred nm. At the same time, the peak at 60 nm shifted to somewhat lower *R*_*H*_ and its height decreased relative to that of the oligomeric peak. *In situ* monitoring of the size distribution indicated a sharp decrease in the height of the aggregate peak between 30 and 40°C, and a corresponding increase in the height of the oligomeric peak, although the peak *R*_*H*_ associated with the aggregates showed no obvious trend with increasing *T* (Fig 2). The initial size distribution changed little on arginine addition, but the increase in the height of the peak at about 8 nm after heat treatment was more marked than in the absence of arginine (Fig 1(b)). The aggregate peak decreased in height between 40 and 50°C in this case, and shifted to lower *R*_*H*_ at higher *T* (Fig 2).

Direct evidence for changes in the morphology of the EMD after heat treatment at pH 2 was obtained by TEM. Nanospheres of up to about 80 nm in diameter and filamentary structures, reminiscent of those reported by Fang *et al*. in recombinant murine amelogenin at low pH [11], were observed in the as-prepared specimens (Fig 1(c)). However, as seen in Fig 1(d), the TEM images were dominated by well-separated nanospheres after heat treatment. The filamentary structures were less in evidence in the presence of arginine, the morphology consisting essentially of nanospheres both before and after heat treatment (Fig 1(e) and 1(f)).

**Fig 3:**
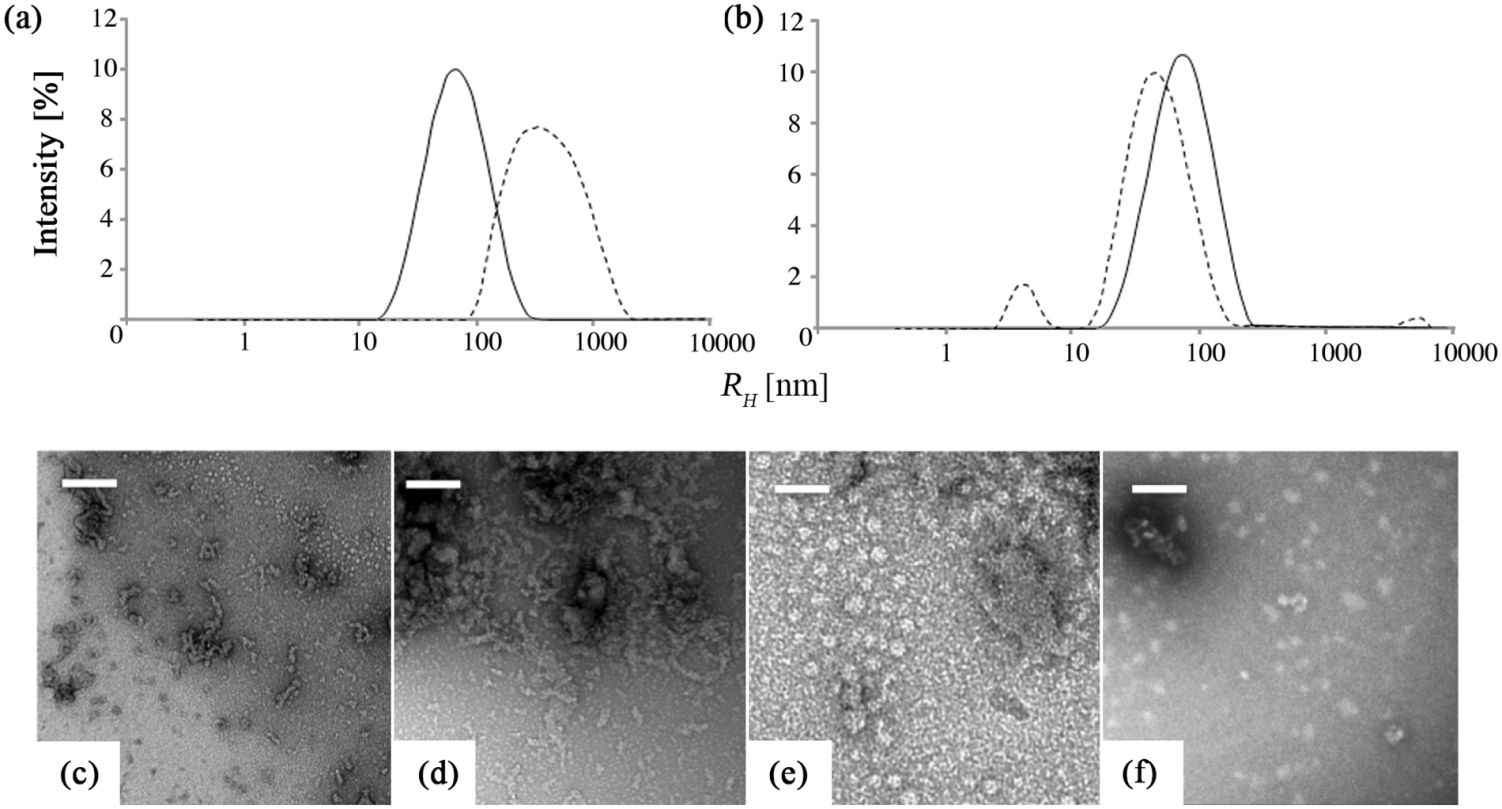
3D-DLS size distributions for EMD at pH 5 and 20°C (a) without and (b) with arginine, before (solid curve) and after (hatched curve) heat treatment at 70°C. TEM micrographs of EMD at pH 5 and 20°C without arginine, (c) before and (d) after heat treatment. TEM micrographs of EMD at pH 5 and 20°C with arginine, (e) before and (f) after heat treatment. The scale bar corresponds to 50 nm in all the images.

As shown in Fig 3(a), the DLS particle size distribution for EMD at pH 5 was initially centered at about 60 nm, but there was no peak at 8 nm and heating led to a marked increase in both the aggregate peak *R*_*H*_ and the aggregate peak height over the whole range of *T* investigated (Fig 2), as reflected by the size distribution after cooling to 20°C. Isolated nanospheres and filamentary structures were visible in the TEM images of the as-prepared specimens (Fig 3(c)). However, extensive, apparently unstructured deposits were observed on the carbon films after heat treatment (Fig 3(d)). As seen from Fig 3(b), arginine addition resulted in little change in the initial particle size distribution at pH 5, but *in situ* monitoring showed the peak to shift to lower *R*_*H*_ as *T* was raised beyond 40°C. An oligomeric peak also appeared at approximately 5 nm at 60°C, so that the overall distribution after heat treatment resembled that observed at pH 2 prior to heat treatment. Moreover, the TEM observations suggested nanospheres once more to dominate the morphology before and after heat treatment at pH 5 in the presence of arginine (Fig 3(e) and 3(f)).

**Fig 4:**
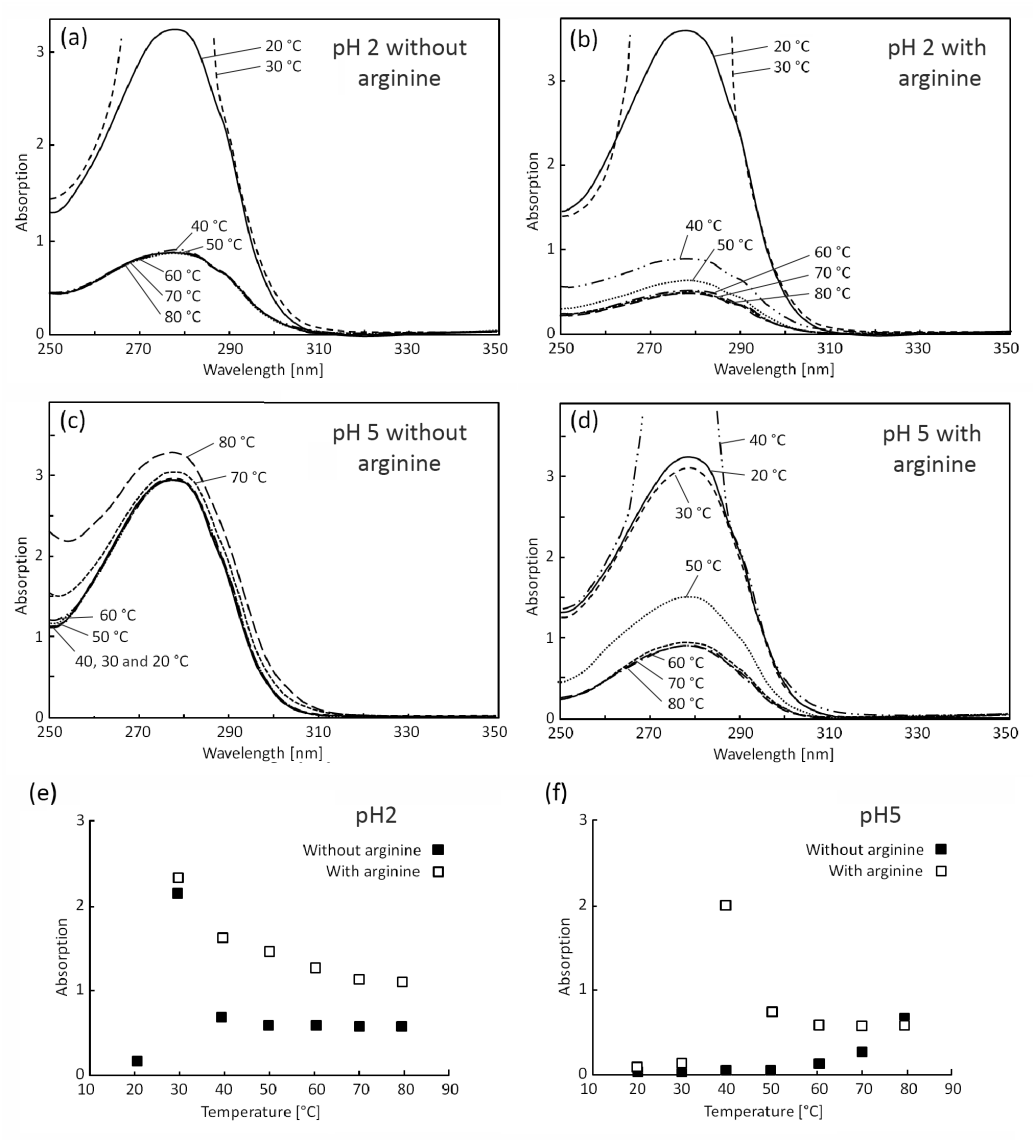
Baseline-subtracted UV-Vis absorption spectra for EMD in BR buffer at pH 2, (a) without and (b) with arginine, and at pH 5, (c) without and (d) with arginine. The corresponding variation with *T* of the baseline signal at 350 nm, where negligible absorption took place, and which was therefore attributed to background scattering, is shown in (e) and (f) for pH 2 and pH 5 respectively.

Fig 4(a) to 4(d) show baseline-subtracted UV-Vis absorption spectra at different pH and *T*. The absorption peak at around 280 nm is characteristic of the hydrophobic tyrosine and tryptophan residues, both of which are present in significant quantities in amelogenin. As shown in Fig 4(a), at pH 2, this peak increased substantially in intensity (hyperchromicity) as *T* was raised from 20 to 30°C. (Indeed at the concentrations of interest here, the absorption in the vicinity of the peak exceeded the instrument detection limit of 7 absorption units.) This may have reflected increased exposure to the solvent of tyrosine and tryptophan present in the hydrophobic core of the EMD proteins, and hence conformational rearrangements during heating, as has been extensively discussed elsewhere in the literature for a range of protein structures [38–44]. It was not possible to use circular dichroism in the present case to confirm exposure of aromatic residues because acetic acid was used as the buffer, as in the manufacture of the EMD-based products that were the focus of this work, and the signal from the acetic acid masked that of the proteins.

The increase in the intensity of the UV-Vis absorption peak at 280 nm was accompanied by a sharp increase in background scattering, assumed to be associated with aggregation (Fig 4(e)). As *T* increased further, however, both the absorption and scattering dropped to much lower levels. Similar behavior was observed at pH 2 in the presence of arginine (Fig 4(b) and 4(e)), but the absorption at 280 nm and the background scattering fell off more gradually at *T* > 30°C. In both cases, absorption spectra obtained after cooling back to 20°C (not shown) were close to those obtained at 20°C before heat treatment.

Contrasting behavior was again observed at pH 5 in the absence of arginine; the absorption peak at 280 nm remained pronounced over the whole range of *T*, and indeed increased in intensity at the highest *T* (Fig 4(c)). Moreover, the background scattering continued to increase, albeit gradually, up to the highest *T* investigated (Fig 4(f)) and was found to remain significantly greater after cooling to 20°C than before heat treatment. On the other hand, arginine addition resulted in qualitatively similar behavior to that observed at pH 2 (Fig 4(d) and 4(f)), consistent with the DLS results, although the sharp increase and subsequent decrease in absorption and scattering with increasing *T* shifted to around 40°C. As for pH 2, spectra obtained on cooling to 20°C after heat treatment in the presence of arginine were close to those obtained at 20°C before heat treatment.

**Fig 5:**
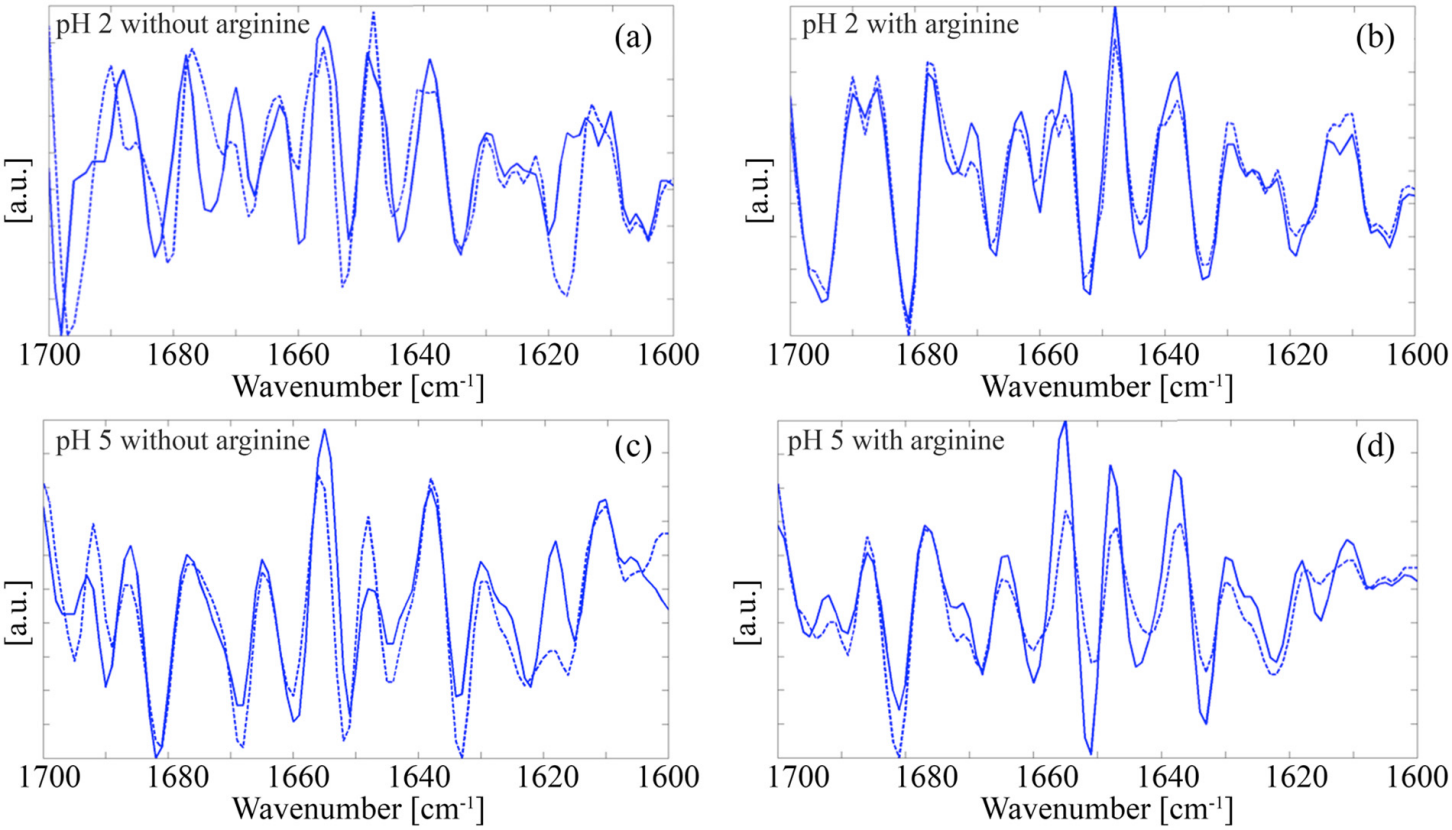
Second derivative FT-IR spectra at 20°C from dried EMD-BR buffer solutions as-prepared (solid curves) and after heat treatment at 80°C (hatched curves): (a) pH 2 without arginine; (b) pH 2 with arginine; (c) pH 5 without arginine; (d) pH 5 with arginine.

Fig 5 shows second derivative FT-IR spectra from the dried EMD-buffer solutions (cf. the images in Figs 1 and 3) in the wavenumber range corresponding to the amide I band, suggesting primarily mixed β-sheet and β-turn structure [45]. Thus, spectra obtained from the specimens prepared at pH 2 (Fig 5(a)), an intramolecular β-sheet peak was visible at 1633 cm^-1^ and the peaks at 1660, 1668 and 1675 cm^-1^ were assigned to β-turns. The peak at 1683 was associated with β-turns/intermolecular β-sheets and there was a further intermolecular β-sheet peak at 1698 cm^-1^. The peaks at 1643 and 1652 cm^-1^ were assigned to random coils and random coils/α-helices, respectively, and the peak at 1620 cm^-1^ was tentatively identified with polyproline II helix structure, based on previous observations of aqueous amelogenin at different pH [12,45].

The β-sheet peak at 1633 cm^-1^ was again prominent in spectra from specimens heat treated at 80°C for 1 hr, but the remaining peaks showed modified intensities and positions. The peak at 1620 cm^-1^ was replaced by a more intense peak at about 1617 cm^-1^, the intermolecular β-sheet peak at 1698 cm^-1^ shifted to about 1697 cm^-1^, and a small additional peak appeared at 1688 cm^-1^. This is significant in that strong peaks in the 1615–1620 cm^-1^ range of the amide I region are typically associated with intermolecular contacts [46]. However, there were also substantial modifications to the β-turn peaks, particularly that at 1675 cm^-1^, which decreased markedly in intensity and shifted to about 1672 cm^-1^. Addition of arginine to the buffer at pH 2 led to similar spectra to those obtained after heat treatment in the absence of arginine. The intensity of the β-turn/intermolecular β-sheet peak at about 1681 cm^-1^ was nevertheless greater in the presence of arginine and the peak at 1617 cm^-1^ was weaker, forming a shoulder to the peak at 1620 cm^-1^, which was also clearly visible in this case (Fig 5(b)). There were little further changes in these spectra after heat treatment.

The FT-IR spectra obtained from the dried EMD-buffer solutions at pH 5 (Fig 5(c)) were distinguished from those for pH 2 by the presence of a relatively strong peak at 1682 cm^-1^, a clear peak at 1690 cm^-1^, and well separated peaks at 1615 and 1623 cm^-1^, a reduction in the intensity and wavenumber of the intermolecular β-sheet peak at 1696 cm^-1^ (1698 cm^-1^ for pH 2), suppression of the β-turn peak at 1675 cm^-1^ and an increase in the intensity of the peak at 1669 cm^-1^ (1668 cm^-1^ for pH 2). Heat treatment resulted in changes in the peak intensities, but no apparent qualitative modifications to the secondary structure, with the exception of the high wavenumber intermolecular β-sheet peaks, which shifted to lower wavenumbers, and the peak at 1615 cm^-1^, which increased markedly in intensity and shifted to about 1616 cm^-1^. The main consequence of addition of arginine to EMD at pH 5 was to modify the β-turn band (Fig 5(d)). Thus, well separated β-turn peaks were visible at 1668 and 1672 cm^-1^, as observed at pH 2 in the presence of arginine, whereas the structure at higher wavenumbers remained similar to that at pH 5 without arginine. Heat treatment in the presence of arginine resulted in changes in the relative intensity of the peaks across the whole of the spectrum, with e.g. a strong decrease in the intensity of the peak at 1615 cm^-1^, but no major shifts in the peak positions.

### Precipitation kinetics from EMD solutions and EMD-PGA gels

**Fig 6:**
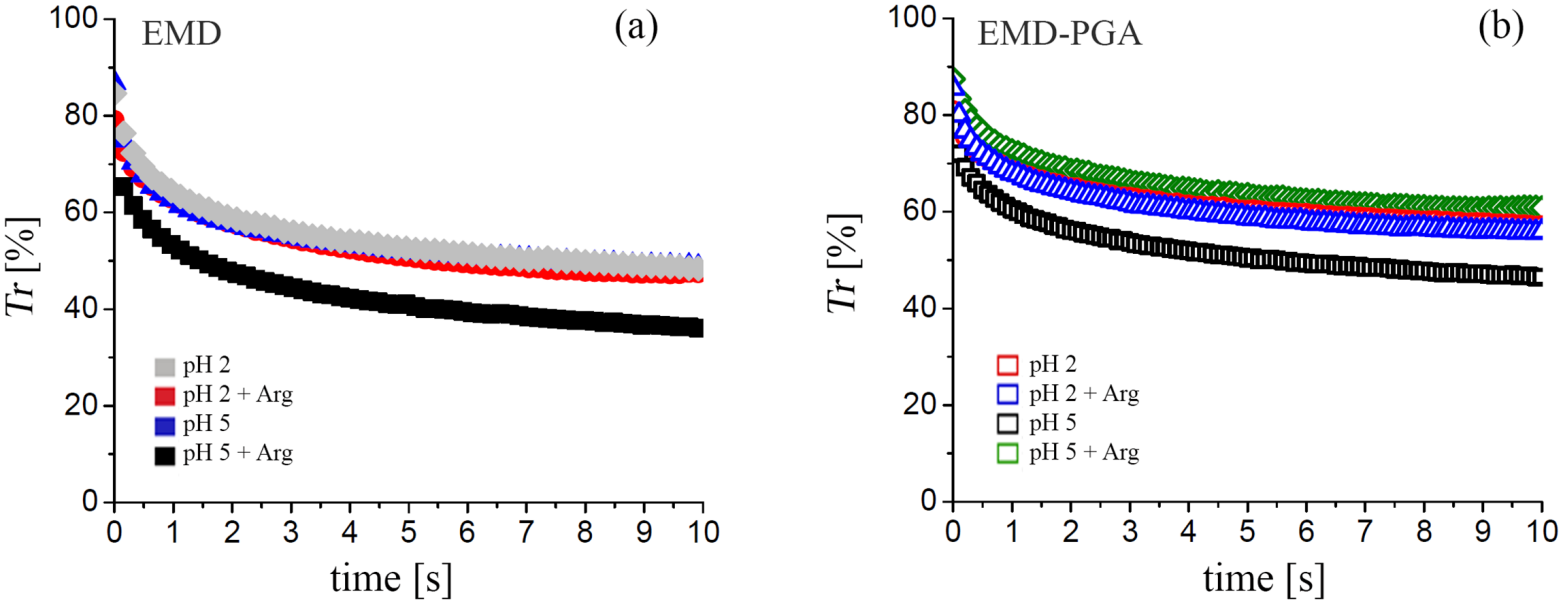
Transmittance, *Tr*, as a function of *t* from stopped-flow measurements on (a) EMD and (b) EMD-PGA in BR buffer (pH 2 or pH 5), with and without arginine, mixed with sodium hydroxide (250 or 50 mM) to raise its pH quasi-instantaneously to 7 at *t* = 0.

Fig 6 shows the transient precipitation kinetics from aqueous EMD and the EMD-PGA gel, as reflected by the stopped-flow measurements of *Tr* as a function of *t*, after raising the pH of the specimens to 7 i.e. close to the EMD isoelectric point (pH 6.8). All the specimens showed similar precipitation dynamics, with the exception of the specimens prepared without arginine at pH 5 (Fig 6(a) and 6(b)). These latter showed significantly greater turbidity (i.e. lower *Tr*) at a given *t*, reflecting a greater tendency to form large precipitates. It follows that formation of complexes with PGA did not modify the response of EMD under conditions representative of its application to damaged periodontal tissue. Furthermore, arginine addition had no significant influence on precipitation from specimens prepared at pH 2, and resulted in similar behavior in specimens prepared at pH 5 to that observed at pH 2.

## Discussion

Even at pH 2, where aqueous amelogenin is widely considered to be in monomeric form at RT, EMD aggregated under the present conditions, forming a mixture of oligomeric structures, filaments and larger spherical aggregates. As *T* was raised through about 30°C, the UV-Vis spectra suggested an increase in the exposure of the hydrophobic residues (possibly associated with a partially folded intermediate), followed by increased aggregation and sedimentation of the larger aggregates. Both UV-Vis and DLS indicated these changes to be largely reversible, although the filamentary morphology observed in the as-prepared specimens at pH 2 was replaced by spherical aggregates after heat treatment. Filamentary structure in amelogenin at low pH may be associated with ionic interactions (e.g. via the acidic residues present in the C-terminal domain of amelogenin [17]), consistent with its relative absence in EMD in the presence of arginine, which is known to interact with negatively charged groups [23]. The presence of filaments may have been reflected by modifications to the high wavenumber β-turn/intermolecular β-sheet bands and the intermolecular β-sheet band in the range 1610 to 1620 cm^-1^ in the FTIR spectra. However, it is concluded from the overall appearance of the spectra that conformational changes were limited during aggregation at low pH, even in the absence of arginine.

An important consideration in the present context is that the BR buffer is a high ionic strength buffer containing phosphate and acetate ions. These may also act as stabilizers for native folded protein structures, and favor salting out according to the Hofmeister series [47]. At high ionic strengths, EMD is expected to be most soluble at low *T*, while the salting out effect may result in agglomeration and precipitation in the folded state at high *T*, as inferred in the present case for pH 2. On the other hand, at low ionic strengths, which favor solubility (salting in), increasing *T* may promote thermally activated unfolding, and ultimately the formation of relatively unstructured aggregates. Hence, competition between thermally-induced unfolding and salting out should depend on the ionic strength of the buffer, as well as pH. In the present case, while the overall concentrations of the BR buffers prepared at pH 2 and 5 were identical, the concentration of the phosphoric acid was lower at pH 5, resulting in a reduction in ionic strength by a factor of approximately 2.

Evidence for more extensive changes in conformation in the reduced ionic strength buffer at pH 5 as *T* was raised included: (i) the apparently amorphous aggregates observed by TEM after heat treatment; (ii) the relative lack of reversibility on thermal cycling as suggested e.g. by the DLS results; (iii) the modifications in the FTIR β-turn band; (iv) the strong intermolecular β-sheet peak at 1616 cm^-1^ in the heat-treated specimens. The FTIR spectra indicated much of the EMD secondary structure to remain intact, even under these conditions. On the other hand, in buffer solutions prepared at pH 8, i.e. well beyond the range of immediate interest for commercial products, EMD was found to become fully denatured, forming large insoluble unstructured aggregates after heat treatment [48].

Arginine addition to the EMD apparently contributed to the salting-out effect at pH 5, resulting in globally similar behavior to that observed at pH 2. Previous studies have shown arginine to act either as an aggregation suppressor or as an aggregation enhancer, depending on its concentration [49]. Thus aggregation is promoted when the arginine concentration is below 700 mM and the protein concentration is greater than 20 mg/mL (here the concentrations were about 500 mM and 29 mg/mL, respectively). Under these conditions, the guanidinium end-group of the arginine amino side-chain has been suggested to act as a bridge, forming H-bonds with the acid residues of separate protein molecules [50, 51]. At relatively low protein concentrations, on the other hand, the guanidinium end-group may interact directly with the aromatic and hydrophobic residues of the proteins, leaving the hydrophilic part of the arginine exposed to the solvent, thus contributing to protein dispersion in aqueous media and shielding intermolecular contacts.

Taken together, these results indicate that the presence of arginine reproduces the benefits of a reduction in pH and/or an increase in the ionic strength of the buffer for the stability of EMD, but without the need to increase the phosphoric acid content, and without adverse consequences for precipitation at physiological pH. Further studies of model systems will be required to validate the hypotheses regarding the underlying mechanisms for this behavior, e.g. systematic investigation of full length amelogenin-arginine interactions under a wider range of conditions, and the influence of other EMD components. Even so, arginine appears highly promising as an additive for EMD and it would be of interest to extend this investigation to look at its influence on the long term evolution in EMD properties and its therapeutic effectiveness under conditions directly representative of dental applications.

## Conclusions

The therapeutic effectiveness of EMD-based products used to treat periodontal defects requires that the EMD be maintained and delivered in its native conformation, i.e. in a form capable of undergoing self-organization to give ordered structures under physiological conditions. This renders problematic any tendency for the EMD to precipitate prematurely in the form of irreversible denatured aggregates during manufacture and/or storage e.g. as a result of heat treatments used for sterilization. The present work has shown that thermal stress may indeed lead to changes in the state of aggregation of EMD in BR buffer under weakly acidic conditions (pH 5), and that these changes are at least partly irreversible on the timescale of the present experiments (heating and cooling times of the order of 10 to 60 min depending on the type of measurement). At lower pH, on the other hand the EMD structure underwent only minor modifications after heating, particularly in the presence of arginine. Moreover, arginine addition to EMD-buffer solutions prepared at pH 5 resulted in a similar structural response to that observed at pH 2, which indicates that arginine may be effective in stabilizing EMD against temperature fluctuations over a relatively wide range of pH. Investigation of the precipitation kinetics of EMD nevertheless suggested arginine not to suppress precipitation from either the buffer solutions or a PGA gel carrier when the pH was raised to 7. These results hence provide evidence that arginine, whose use in products for dental treatment is already validated, has the potential to improve the stability of EMD-products for the treatment of periodontal defects during manufacture and storage, without affecting the ability of current EMD-carrier systems to deliver the EMD to the defect site on exposure to physiological conditions.
